# Extra-Lobar Lung Sequestration Infarction That Caused Sudden Back Pain: An Adult Case of Surgical Resection

**DOI:** 10.70352/scrj.cr.24-0032

**Published:** 2025-01-31

**Authors:** Noriyoshi Sawabata, Isao Arai, Hisanori Hatano, Takashi Ito, Yuko Fukumoto, Takayuki Minoji, Kotaro Muranishi, Takahiko Nishigaki, Ken Konishi, Kazuyuki Matsushita, Kazunori Nakaguchi, Sadayuki Doi, Keishi Sugimoto

**Affiliations:** 1 Department of Surgery, Kawanishi City Medical Center, Kawanishi, Hyogo, Japan; 2 Department of Pathology, Kawanishi City Medical Center, Kawanishi, Hyogo, Japan

**Keywords:** extra-lobar pulmonary sequestration, infarction, back pain, adult, surgery

## Abstract

**INTRODUCTION:**

Ischemia in extra-lobar pulmonary sequestration is rare and mostly occurs in childhood; it is uncommon in adults but can be progressive, necessitating surgical removal.

**CASE PRESENTATION:**

A 37-year-old woman experienced sudden onset severe back pain and was initially diagnosed with pneumonia. Computed tomography revealed a 4.5 cm mass on the diaphragm and rapidly increasing pleural effusion. Emergency surgery confirmed a black mass that adhered to the diaphragm, which could be bluntly detached, but one was fixed in a cord shape that was detached by an energy device. Pathology showed significant hemorrhage and tissue destruction in extra-lobar pulmonary sequestration.

**CONCLUSIONS:**

Extra-lobar pulmonary sequestration infarction, which develops suddenly with pain as the main complaint, is rare and mainly seen in early childhood but can also occur in adults.

## Abbreviations

CRP
: C-reactive peptide
CT
: computed tomography
HU
: Hounsfield unit
LDH
: lactate dehydrogenase

## INTRODUCTION

Sudden chest pain based on the lungs is due to inflammatory reactions caused by organ ischemia owing to pulmonary infarction, pulmonary lobar torsion, pulmonary thromboembolism, etc.^1,2)^. The rare occurrence of the same condition in extra-lobar lung sequestration, which occurs mostly in childhood^3–5)^, occurred in this adult case.

## CASE PRESENTATION

**Fig. 1:**
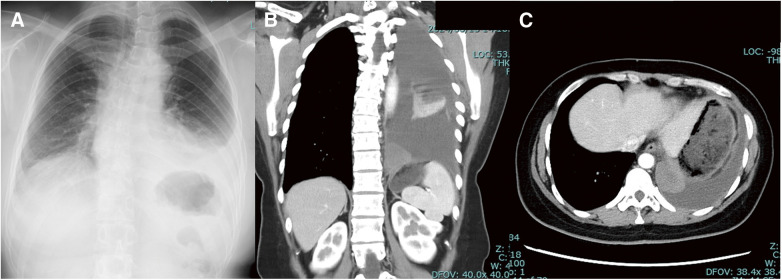
Radiographic findings before surgery. Left pleural effusion and left atelectasis are noted (**A**). A mass lesion with a maximum diameter of 4.5 cm is observed on the left side at the Th11 level (**B**). The contrast-enhanced effect of the mass is rarely observed from the arterial phase to the late phase. It is presumed that the area around the mass is located within the mediastinum, with a thickened pleura (**C**).

A 37-year-old woman had a history of resection of a huge leiomyoma of the uterus. Only a magnetic resonance image of the abdomen was performed before the surgery, but this examination did not depict a lesion in the chest cavity. She suddenly felt severe back pain. She went to a nearby practitioner, was diagnosed with pneumonia, and was given antibiotics and anti-inflammatory drugs. However, since the symptoms did not improve, she visited a nearby hospital and was referred to a regional medical core hospital due to an elevated white blood cell count (14.9 × 10^3^/mL) and an elevated C-reactive peptide (CRP) value (18.1 mg/dL). A simple chest computed tomography (CT) examination indicated a flat mass on the diaphragm with a maximum diameter of about 4.5 cm near the vertebral body, along with a small amount of pleural effusion. Although both the white blood cell count (10.4 × 10^3^/mL) and the CRP value (2.8 mg/dL) improved after 1 week of administration of antibiotics and nonsteroid anti-inflammatory drugs, a rapid increase in the fluid was observed on radiographic examination (**Fig. 1**), and back pain persisted. From pleural effusion accompanying chest pain, we considered pathophysiology such as empyema, hemothorax, and pleurisy. The inflammatory findings of the blood test improved; SpO_2_ was normal at 97% in room air, and there was no elevation of lactate dehydrogenase (LDH), but the pain did not improve. A contrast-enhanced chest CT revealed an increasing pleural effusion (39 Hounsfield unit [HU]) that required differentiation from bleeding, and so emergency surgery was performed for diagnostic and therapeutic purposes. Although the lesion was not contrast-enhanced by a CT scan, the possibility of occlusion of the feeding artery could not be ruled out if the lesion was extra-lobar lung sequestration.

**Fig. 2:**
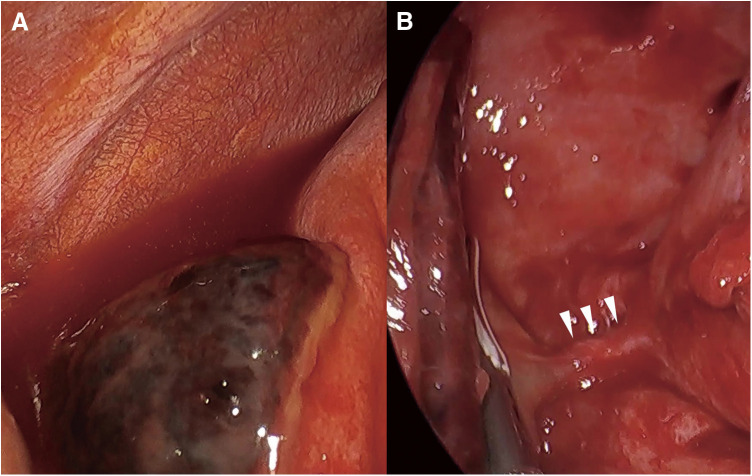
Surgical findings. A triangular tortoiseshell-patterned black object is present in contact with the diaphragm, and noncoagulable serum pleural effusion is observed around it (**A**). When the adhesion between the lesion and the diaphragm was bluntly detached and departed to the cephalic side, a white cord-like object (arrowhead) several millimeters in diameter, which seemed to originate from the descending aorta, reached the lesion (**B**).

We performed thoracoscopic surgery with a 5-mm scope in the right-sided decubitus position under one-lung ventilation. About 700 mL of noncoagulable bloody pleural effusion was drained. A black mass adhered to the diaphragm (**Fig. 2A**); it could be bluntly detached, but one was fixed in a cord shape (**Fig. 2B**) and detached by an energy device. The diameter of the cord was only a few millimeters, and it was excised using an energy device in the very vicinity of the fractionated lung. It was judged to be an intrathoracic lesion based on contrast-enhanced CT findings, but it was in wide contact with the diaphragm, and there was a possibility of combined resection. A prosthetic membrane was prepared on-site; however, the surgery was completed under video-assisted thoracic surgery without using it. After confirming that there was no bleeding, a 20Fr silicon thoracic tube was inserted from the middle axillary line at the ninth intercostal space, and the chest was closed. The patient had a good postoperative course; the chest drain was removed on the second postoperative day, and she was discharged on the fourth postoperative day.

**Fig. 3:**
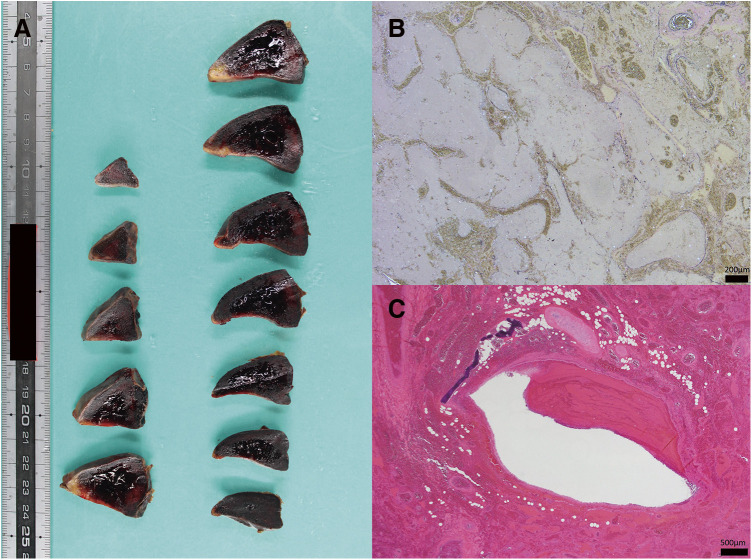
Pathological finding of the lesion. Grossly, it is a lesion with a high degree of hemorrhage and significant tissue destruction (**A**). The outermost layer is covered with a fibrous capsule, and adipose tissue, along with fibrous granulation tissue, is found directly below it. Blood vessels and alveolar-like cystic structures that show meandering and branching abnormalities are observed inside (**B**, Elastica Van Gieson stain). Bronchial structures with cartilage are also present (**C**, hematoxylin–eosin stain). It is challenging to search for the pathological history of the cord due to thermal degeneration that enters the removed lesion.

Grossly, it is a lesion with a high degree of hemorrhage and significant tissue destruction (**Fig. 3A**). Malformed diseases such as extra lobar pulmonary sequestration are lesions that have caused infarction, and no malignant findings exist (**Fig. 3B, C**). It was challenging to search for pathological history due to thermal degeneration that entered the removed lesion.

## DISCUSSION

Our study highlights the rare occurrence of extralobar pulmonary sequestration infarction in adults, a condition primarily observed in childhood. Epidemiological data indicate that pulmonary sequestration affects approximately 0.15%–1.7% of patients, with extralobar pulmonary sequestration accounting for 15%–25% of these cases^6)^. The incidence of extralobar pulmonary sequestration infarction in adults is particularly rare and warrants detailed examination.

In cases of extralobar pulmonary sequestration, the visceral pleura is not continuous with that of the normal lung, and the blood supply is systemic, typically originating from the thoracic or abdominal aorta in about 80% of cases^7)^. However, when torsion or obstruction of the aberrant artery occurs, as in our case, the contrast effect may be absent on imaging, complicating diagnosis.

The differential diagnosis for pulmonary sequestration includes conditions such as pulmonary infarction, pulmonary embolism, and lobar torsion. It is critical to differentiate these conditions based on clinical presentation and imaging findings. For instance, CT HU can help distinguish between hemorrhagic and nonhemorrhagic pleural effusions, aiding in accurate diagnosis^8)^.

Previous reports have noted the potential for malignant transformation and recurrent infections in pulmonary sequestration discovered later in life, underscoring the importance of elective resection^9)^. Our case demonstrates that surgical intervention is often necessary to prevent severe complications, even in the absence of symptoms. The lack of contrast enhancement in our patient’s imaging suggested vascular occlusion, leading to the decision for emergency surgery.

Comparing pediatric and adult cases of pulmonary sequestration, it is evident that adult cases may present with atypical symptoms and complications, such as our patient’s severe back pain and pleural effusion. Pediatric cases often show congenital anomalies, while adult cases may result from long-term undiagnosed pulmonary sequestration with gradual symptom development.

Symptoms such as sudden chest or back pain, along with imaging findings of pleural effusion and nonenhancing masses, should raise suspicion for pulmonary sequestration. Our case emphasizes the importance of considering pulmonary sequestration in differential diagnoses for adults presenting with these symptoms despite its rarity.

Surgical approaches for managing pulmonary sequestration include thoracoscopic surgery, as demonstrated in our case. The decision to perform surgery should be based on the severity of symptoms and the potential for complications. In our patient, thoracoscopic surgery successfully removed the infarcted lung tissue and alleviated symptoms, highlighting the efficacy of this minimally invasive approach.

## CONCLUSIONS

While extralobar pulmonary sequestration infarction is rare in adults, it should be considered in the differential diagnosis of sudden chest or back pain with pleural effusion. Detailed imaging and prompt surgical intervention are crucial for effective management and improved patient outcomes.

## ACKNOWLEDGMENTS

The authors thank Dr. Kaoru Kobayashi for the radiographic diagnosis.

## DECLARATIONS

### Funding

There is no funding to be declared.

### Authors’ contributions

Article writing, N.S.

Data collection, N.S.

Clinical practice, N.S., I.A., H.H., T.I., Y.F., T.M., K.M., T.N., K.K., K.M., K.N., S.D., and K.S.

Proofing, N.S., I.A., H.H., T.I., Y.F., T.M., K.M., T.N., K.K., K.M., K.N., S.D., and K.S.

All authors agree to take responsibility for all aspects of the study.

### Availability of data and materials

There is no dataset supporting the conclusions of this article.

### Ethical approval and consent to participate

The ethical committee of Kawanishi City General Hospital has approved publishing this case report.

### Consent for publication

Consent was obtained from the patient for publication.

### Competing interests

There are no competing interests.
